# Discovery and analysis of a new class of triterpenes derived from hexaprenyl pyrophosphate

**DOI:** 10.1016/j.engmic.2022.100035

**Published:** 2022-07-08

**Authors:** Dan Hu

**Affiliations:** Institute of Traditional Chinese Medicine & Natural Products, College of Pharmacy / Guangdong Province Key Laboratory of Pharmacodynamic Constituents of TCM and New Drugs Research / International Cooperative Laboratory of Traditional Chinese Medicine Modernization and Innovative Drug Development of Ministry of Education (MOE) of China, Jinan University, Guangzhou 510632, China

**Keywords:** Biosynthesis, Non-squalene triterpenes, Terpene cyclase, Cyclization mechanisms

## Abstract

Triterpenes are derived from squalene or oxidosqualene. However, a new class of triterpenes derived from hexaprenyl pyrophosphate has been recently discovered, formed by a new family of chimeric class I triterpene synthases. The cyclization mechanisms of triterpenes were elucidated by isotopic labeling and protein structural analyses, which helps understand the biosynthesis of triterpenes in nature.

## Graphical abstract

**Figure:**
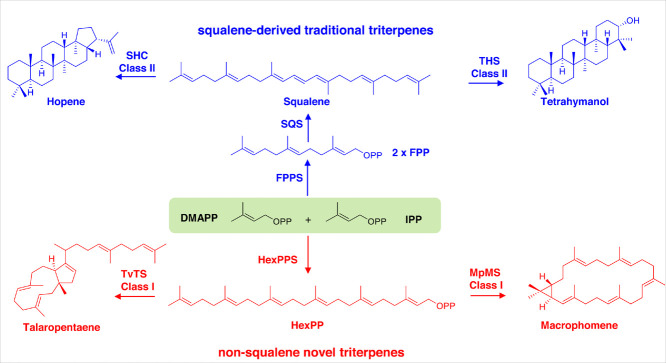

## 1 Introduction

**Fig. 1:**
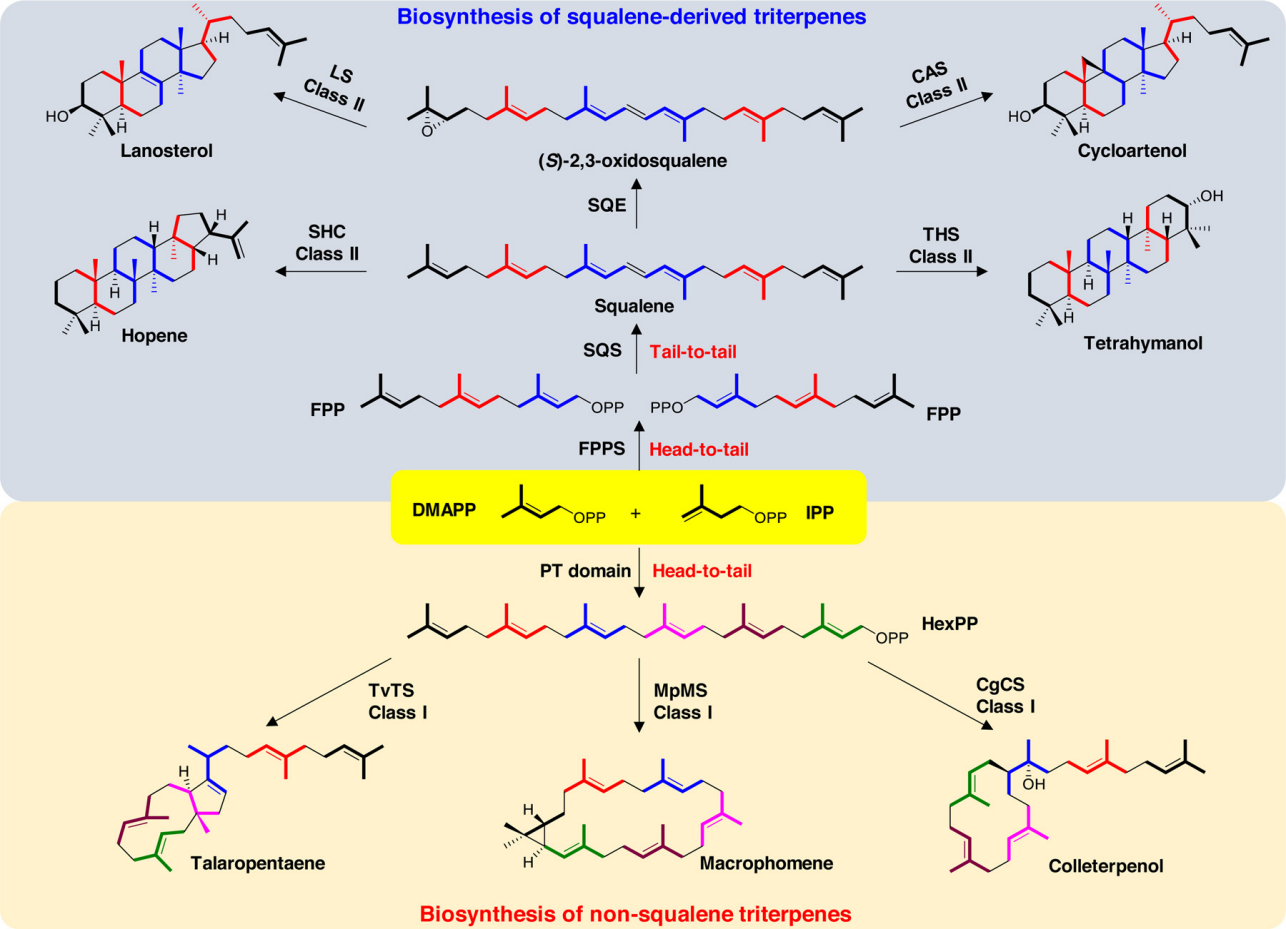
Biosynthesis of triterpenes. Biosynthesis of squalene-derived triterpenes are initiated by condensation of one DMAPP with two IPP into FPP. Subsequent tail-to-tail coupling of two FPP molecules by SQS afford squalene, which is oxidized to (S)-2,3-oxidosqualene by squalene epoxidase (SQE). Cyclization of squalene and oxidosqualene by a large group of triterpene synthases generates diverse triterpene skeletons. In contrast, one DMAPP and five IPP can be head-to-tail condensed to HexPP by the PT domains of the new type of triterpene synthases and cyclized into non-squalene triterpenes.

Triterpenes are the largest classes of natural products widely distributed in nature, including animals, plants, fungi, and bacteria. About 20,000 triterpenes have been discovered in nature [14,17] and possess a variety of biological functions [5]. Triterpenes are produced from two common precursors, squalene and its epoxied analog oxidosqualene, formed by squalene synthase (SQS)-mediated tail-to-tail coupling of two farnesyl pyrophosphate (FPP) molecules (Fig. 1). Therefore, squalene and oxidosqualene do not contain pyrophosphate groups, so the subsequent cyclization proceeds *via* substrate protonation rather than pyrophosphate release.

Triterpene synthases belong to a class II terpene synthases (TSs), containing a conserved DXDD motif for the protonation of the terminal olefin or epoxide of squalene and oxidosqualene. The representative enzymes include squalene hopene cyclase (SHC), tetrahymanol synthase (THS), lanosterol synthase (LS), and cycloartenol synthase (CAS) (Fig. 1). [8,13]. In contrast, the class I terpene cyclases (TCs) possess two distinct conserved motifs, DDXX (D/E) and NSE/DTE, and initiate terpene cyclization by releasing the pyrophosphate group [3]. Many examples of class I TCs have been shown to convert linear prenyl chains with different lengths (C_10_ to C_25_) into various polycyclic terpenes [4,12]. Nevertheless, no class I TC can convert hexaprenyl diphosphate (HexPP; C_30_) into triterpenes, although, HexPP is widely distributed in nature [9,15].

A collaborative study between Liu from Wuhan University in China, Abe from the University of Tokyo in Japan, and Dickschat from the Bonn University in Germany revealed a novel class of chimeric fungal class I triterpene synthases, capable of producing HexPP from DMAPP and IPP, and subsequently cyclize HexPP into non-squalene triterpenes (Fig. 1) [16]. Using the yeast-based genome mining platform [1], Liu's team discovered two fungi-derived types I chimeric TSs, TvTS and MpMS, the bifunctional enzymes harboring an N-terminal TC domain and C-terminal prenyl transferase (PT) domain. Although chimera TSs have been frequently reported in fungi [2,18], TvTS and MpMS can convert DMAPP and IPP into triterpene skeletons. *In vivo* gene activation and *in vitro* enzymatic assay showed that the PT domains catalyze the head-to-tail condensation of one DMAPP with five IPP to form HexPP, and the TC domains of TvTS and MpMS then catalyze the cyclization of HexPP to talaropentaene and macrophomene, respectively (Fig. 1). The final conformation and cyclization mechanism of talaropentaene (**1**) and macrophomene (**2**) were determined by Dickschat's team using an *in vitro* isotope feeding experiments, which follows a C1-III-IV cyclization mode and an unprecedented C1-VI cyclization mode and represents the largest macrocycle discovered in terpenes, respectively.

Next, Abe's team investigated the catalytic mechanism of TvTS and MpMS by determining the crystal structure of the TC domain of TvTS in combination with site-directed mutagenesis. They found that TvTS and MpMS adopt different strategies to accommodate HexPP for cyclization. TvTS evolves an extra tunnel based on the ball-shaped cavity of class I diterpene synthases, while MpMS provides a larger cavity for HexPP uptake. In addition, the binding mode between PT and TC domains of MpMS was determined by cryo-electron microscopy (cryo-EM), which is significantly distinct from that of chimeric diterpene synthase (PaFS) [6]. Finally, using AlphaFold2 prediction and docking analysis, Liu's team efficiently screened and identified two other triterpene synthases, CgCS and PTTC074, which convert HexPP into colleterpenol (**3**) (Fig. 1), suggesting the wide distribution of non-squalene triterpene synthases in nature.

Terpenes are the most chemically and structurally diverse natural products [3]. The discovery by Liu, Dickschat, and Abe has dramatically expanded our understanding of TCs’ great potential and power. Recently, many uncanonical TCs have been identified, including the UbiA superfamily of prenyltransferases acting as TCs [10,11], the class I TCs serving as prenyltransferases [7], and the novel chimeric triterpene synthases described in this study [1]. These reports together indicate the great potential of TCs to generate massive metabolism treasure. Deep mining combined with artificial intelligence technology will discover more unclassical and intriguing biosynthetic transformations. Overall, these findings described in this study are eye-opening and enhanced our understanding of terpene biosynthesis in nature.

## Declaration of Competing Interest

The authors declare that they have no known competing financial interests or personal relationships that could have appeared to influence the work reported in this paper.
